# Supplementary material for: RNA G-Quadruplex within the 5′-UTR of FEN1 Regulates mRNA Stability under Oxidative Stress
Source: Antioxidants (Basel). 2023 Jan 26;12(2):276. doi: 10.3390/antiox12020276 (PMC9952066; doi:10.3390/antiox12020276)
Supplement: Supplementary file 1 [file antioxidants-12-00276-s001.zip › antioxidants-2112544-supplementary.pdf]

RNA G-quadruplex within the 5'-UTR of FEN1 regulates mRNA stability under oxidative stress

Ying Ma[1], Yang Yang[1,2], Jingyu Xin[1], Lingfeng He[1], Zhigang Hu[1], Tao Gao[1], Feiyan Pan[1]* and Zhigang Guo[1]*

[1] Jiangsu Key Laboratory for Molecular and Medical Biotechnology, College of Life Sciences, Nanjing Normal University, 1 Wen Yuan Road, Nanjing, 210023, China

[2] College of Life Science, Northeast Agricultural University, Harbin 150030, China.

Supplemental Information

The following file contains supplementary material for the paper "RNA G-quadruplex within the 5'-UTR of FEN1 regulates mRNA stability under oxidative stress".

This file is composed of:

- Supplementary tables (2 tables)

Table S1. The PQSs of FEN1 5'UTR regions.

Name	location	Sequence
r-PQS1	+55~+108	C**GGG**UGUAGA**GGG**AGCAG**GGG**CCUGC**GGG**GACCUGGUGU**GGG**UGGAGU**GGGG**AC
r-PQS2	+55~+86	C**GGG**UGUAGA**GGG**AGCA**GGGG**CCUGC**GGGG**AC
r-PQS3	+64~+108	A**GGG**AGCAG**GGG**CCUGC**GGG**GACCUGGUGU**GGG**UGGAGU**GGGG**AC
r-PQS4	+71~+108	AG**GGG**CCUGC**GGG**GACCUGGUGU**GGG**UGGAGU**GGGG**AC
r-PQS5	+55~+97	C**GGG**UGUAGA**GGG**AGCAG**GGG**CCUGC**GGG**GACCUGGUGU**GGG**U
r-PQS6	+64~+97	A**GGG**AGCAG**GGG**CCUGC**GGG**GACCUGGUGU**GGG**U
rG4m1		AG**AAA**CCUGC**GGG**GACCUGGUGU**GGG**UGGAGU**GGGG**AC
rG4m2		AG**GGG**CCUGC**AAA**GACCUGGUGU**GGG**UGGAGU**GGGG**AC
rG4m3		AG**GGG**CCUGC**GGG**GACCUGGUGU**AAA**UGGAGU**GGGG**AC
rG4m4		AG**GGG**CCUGC**GGG**GACCUGGUGU**GGG**UGGAGU**AAAA**AC
^{oxo}G20		AG**GGG**CCUGC**GGG**GACCUGGUGU**GGG**UGGAGU**G**^{oxo}**GGG**AC

Table S2: The primer sequences.

Name	Sequence
qPCR-FEN1-F	5'- ATCCCTTATCTTGATGCACCC -3'
qPCR-FEN1-R	5'- AGTCAGGTGTCGCATTAGC -3'
qPCR-β-actin-F	5'- ACATCCGCAAAGACCTGTAC -3'
qPCR-β-actin-R	5'- TGATCTTCATTGTGCTGGGTG -3'
Sh-hnRNPA1-F	5'- GATCCGGGATGGCTATAATGGATTTGTTCAAGAGACAAATCCATTATAGCCATCCCTTTTTTGGAAA -3'
Sh-hnRNPA1-R	5'- AGCTTTTCCAAAAAAGGGATGGCTATAATGGATTTGTCTCTTGAACAAATCCATTATAGCCATCCCG -3'
Sh-NC-F	5′- GATCCGCACAAGCTGGAGTACAACTTCAAGAGAGTTGTACTCCAGCTTGTGCTTTTTTGGAAA -3′
Sh-NC-R	5′- AGCTTTTCCAAAAAAGCACAAGCTGGAGTACAACTCTCTTG AAGTTGTACTCCAGCTTGTGCG -3′
